# Comparing intracorporeal mechanical anastomosis vs. hand-sewn esophagojejunostomy after total laparoscopic gastrectomy for esophagogastric junction cancer: a single-center study

**DOI:** 10.1186/s12957-023-02889-y

**Published:** 2023-01-17

**Authors:** Jiang Peng Wei, Wei Dong Wang, Xi Sheng Yang, Xin Guo, Xiao Hua Li, Gang Ji

**Affiliations:** Department of Gastrointestinal Surgery, Xijing Hospital, Air Force Military Medical University, Xi’an, 710032 Shaanxi China

**Keywords:** Gastric cancer, Total laparoscopic gastrectomy, Laparoscopy, Hand-sewn, Digestive tract reconstruction

## Abstract

**Objective:**

This study aimed to compare the effects of continuous hand-sewn esophagojejunostomy with barbed suture and mechanical anastomosis in total laparoscopic gastrectomy for esophagogastric junction cancer.

**Materials and methods:**

The clinical data of 60 patients who underwent total laparoscopic total gastrectomy from January 2020 to October 2021 were collected retrospectively. Baseline data and short-term surgical results of patients in the hand-sewn anastomosis (*n* = 30) and mechanical anastomosis (*n* = 30) groups were analyzed.

**Results:**

No significant differences were detected in the baseline data between groups. Meanwhile, the hand-sewn group had a shorter anastomosis time (21.2 ± 4.9 min vs. 27.9 ± 6.9 min, *p* < 0.001) and a decreased operation cost (CNY 70608.3 ± 8106.7 vs. CNY 76485.6 ± 3149.9, *p* = 0.001). The tumor margin distance in the hand-sewn group was longer than in the mechanical group (2.7 ± 0.4 cm vs. 2.2 ± 0.75 cm, *p* = 0.002). In esophagojejunostomy anastomosis, the distance between the jejunal opening and jejunal stump in the hand-sewn group was significantly shorter than that in the mechanical group (2.2 ± 0.54 cm vs. 5.7 ± 0.6 cm, *p* < 0.001). No significant difference was detected in the incidence of postoperative anastomotic complications.

**Conclusion:**

The continuous hand-sewn anastomosis with barbed suture in total laparoscopic gastrectomy for esophagogastric junction cancer is practical, safe, and cost-effective. It is also an effective supplementary technique for mechanical anastomosis.

**Supplementary Information:**

The online version contains supplementary material available at 10.1186/s12957-023-02889-y.

## Introduction

Totally, laparoscopic total gastrectomy (TLTG) has been increasingly used to treat upper or mid-third gastric cancer because it has earlier recovery and is less invasive [1]. Esophagojejunostomy is the most crucial process of the entire TLTG procedure. When performing esophagojejunostomy, the linear stapler method is widely adopted due to its simplicity compared to the circular stapled method, including the overlap and functional methods [2]. Since it is convenient to complete laparoscopic gastroenteric anastomosis with a machine, it is widely used in clinical practice. However, anastomosis complications cannot be avoided, and the incidence of esophagojejunostomy leakage can be as high as 14.5% [3]. Thus, to reduce the bleeding and leakage of anastomosis, a manual suture is used to strengthen the anastomosis in most cases. Additionally, to avoid anastomotic stenosis, it is also a good choice to use a manual suture to close the common opening when using the linear stapler for side-to-side anastomosis [4, 5]. Hence, mechanical anastomosis still cannot replace hand-sewn anastomosis. Compared with mechanical anastomosis, hand-sewn anastomosis has fewer clinical applications and higher requirements for good suture techniques. Nevertheless, the advantages associated with this method include low-cost operation in a narrow space, a good surgical field of view to avoid excessive traction, and easy obtainment of the pathology of the esophageal cutting edge before anastomosis [6]. Hand-sewn anastomosis has been reported to be a safe and feasible procedure [6–10], but the clinical research remains insufficient. Although many laparoscopic suture methods are available, more simple, reliable, and repeatable anastomotic methods still need to be explored. With the development of barbed sutures technology in recent years, our institution has explored TLTG for gastric cancer using barbed sutures for totally hand-sewn anastomosis, simplifying the process and reducing the difficulty of the suture to enhance practicability. Herein, we report our experience with this procedure.

## Materials and methods

### Clinical data

**Fig. 1:**
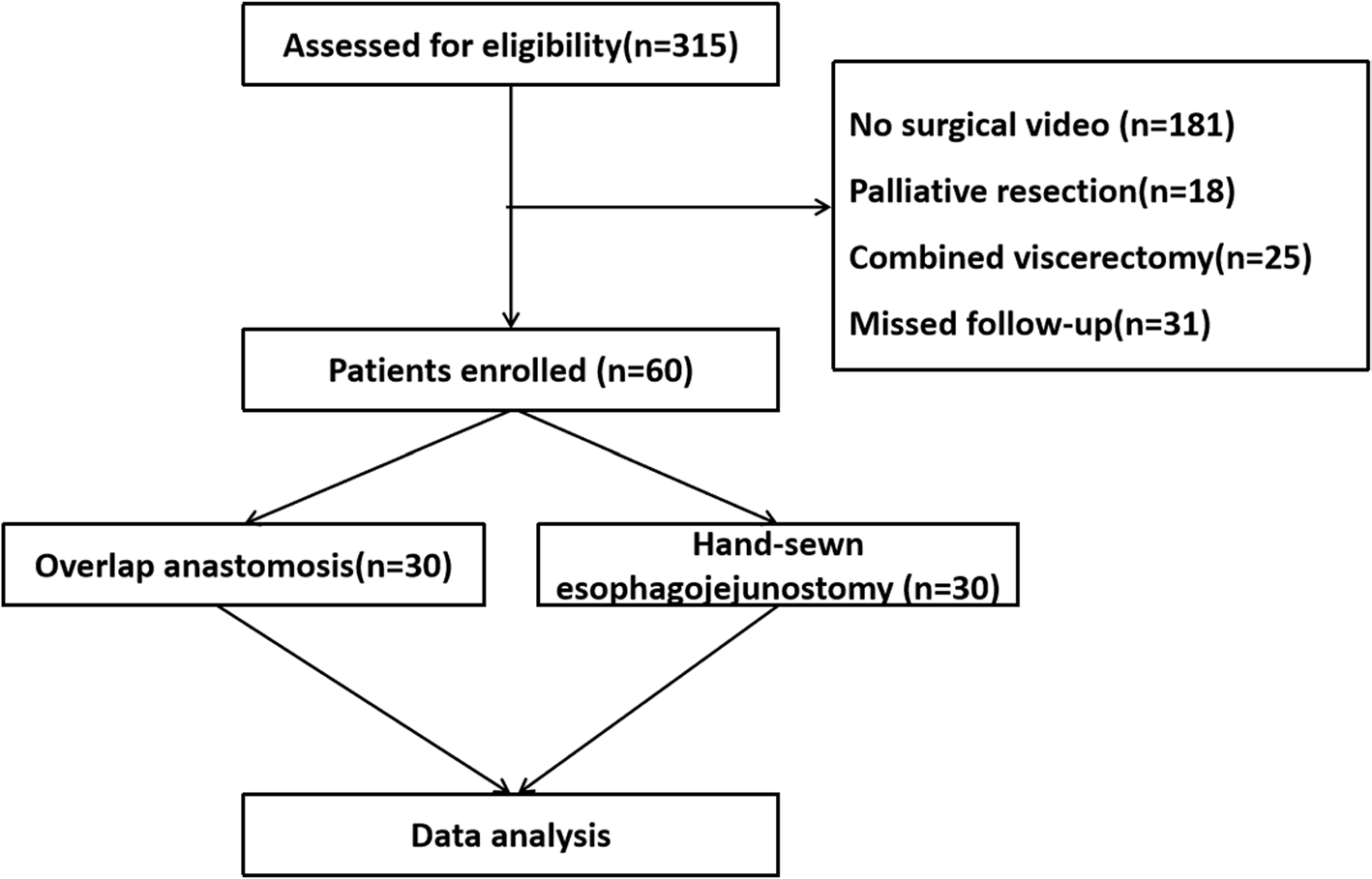
The flowchart of patient selection

We collected the clinical data of 60 patients who underwent TLTG + D2 lymph node dissection for gastric cancer at the Department of Gastrointestinal Surgery of the First Affiliated Hospital of Air Force Military Medical University from January 2020 to October 2021. Among them, 30 patients underwent esophagojejunostomy with continuous hand-sewn. The other 30 patients undergoing mechanical anastomosis comprised the control group. The inclusion criteria were (1) TLTG + D2 lymph node dissection for gastric cancer; (2) digestive tract reconstruction was performed by Roux-en-Y anastomosis, intracorporeal mechanical anastomosis, and hand-sewn esophagojejunostomy with 3-0 barbed suture; (3) patients signed the informed consent; (4) the operator should have complete at least 100 experiences of laparoscopic distal or total gastrectomy. The exclusion criteria were (1) palliative resection or combined viscerectomy for gastric cancer; (2) complicated with underlying severe diseases and unable to continue related treatment procedures; (3) complicated with severe mental illness and unable to complete the treatment process; (4) patient refused to cooperate; (5) no surgical videos. We followed the 8th American Joint Committee on Cancer (AJCC) TNM staging system guideline for staging (Fig. 1).

### Operation method

**Fig. 2:**
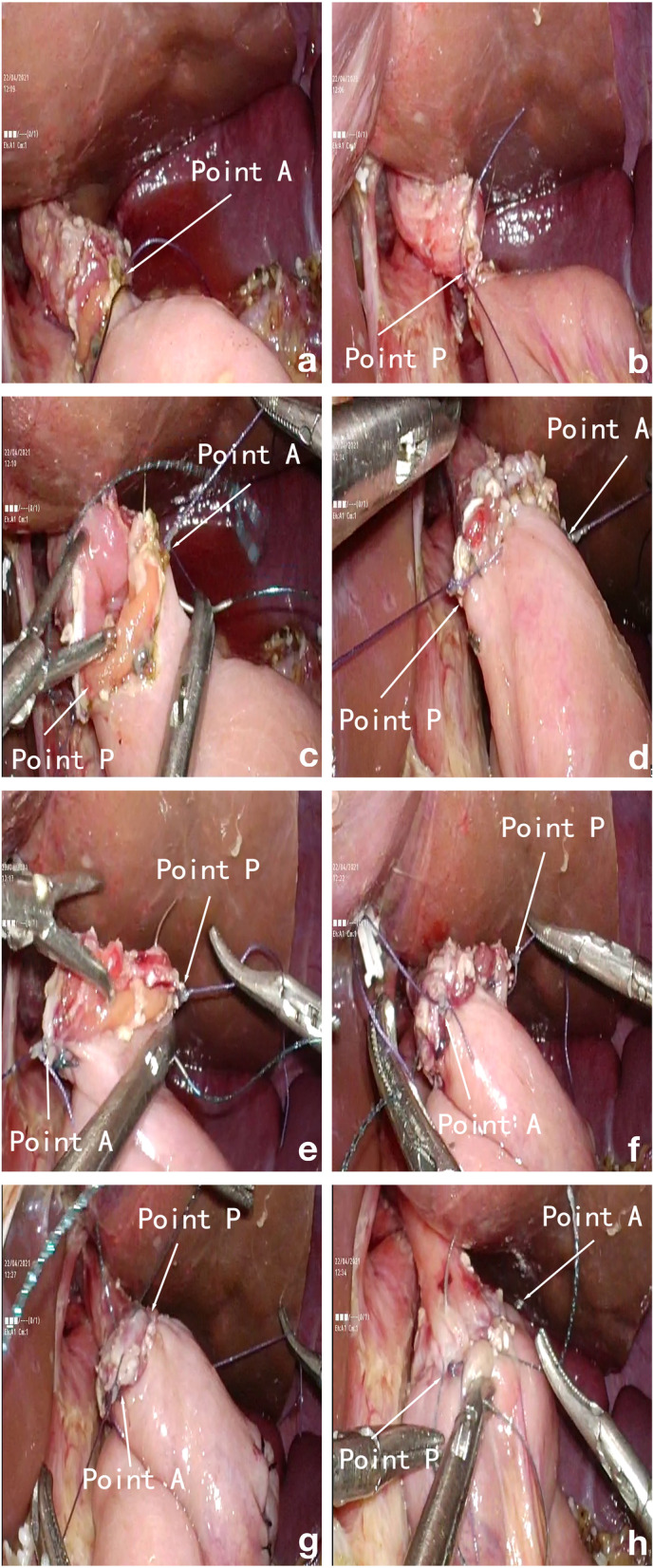
**A** Point P suture. **B** Point A suture. **C** The right wall was fully sutured from point A to point P. **D** Full layer suture to point P to complete the right wall suture. **E** Rotate the point A suture counterclockwise to the right rear side and point P to the left front side, and start full-thickness suture from point P. **F** Complete the full-thickness suture of the left wall. **G** Left wall anastomotic stoma with enhanced embedding suture of seromuscular layer. **H** Enhanced embedding suture of seromuscular layer of right wall anastomosis

First, the patient was placed in the supine position with the lower limbs separated, and general anesthesia was performed by endotracheal intubation. The upper abdomen five-hole method was used to establish pneumoperitoneum, and the laparoscope was placed through the umbilical puncture hole. The other puncture holes, located under the costal margin in the axillary front and between the umbilical hole and the costal margin, were symmetrically distributed. The jejunum was severed with a linear cutting closure device 15 cm from the Treitz ligament. The input and output loops jejunum were anastomosed with a 45-mm linear cutting closure device at the sub umbilical incision. The common opening and mesangial hiatus were closed by 3-0 absorbable suture, and the residual jejunum was sutured with intermittent embedding. An incision of about 0.5 cm was opened by an electric knife at about 2–3 cm near the stump in hand-sewn group, 4–5 cm in mechanical anastomosis group. Pneumoperitoneum was re-established when the intestinal tube was inserted into the abdominal cavity. Side-to-side anastomosis of the left posterior wall of the esophagus and the anterior wall of the jejunum was performed. After the esophagojejunostomy was completed by a 45-mm linear cutting and closing device, the laparoscopic mechanical method was used to perform 15-cm-long 3-0 barbed sutures (V-Loc 180; Covidien, Mansfield, MA, USA) to close common openings, as previously described [7]. The manual suture method was used to dissect the esophageal stump with an ultrasonic knife and disinfect it with iodophor gauze: (1) the operator and the first assistant replaced each other. The operator was on the right, and the first assistant was on the left. A 3-0 absorbable suture was used in the middle of the posterior wall of the anastomotic end of the esophagus jejunum, defined as point P (Fig. 2A). About 5 cm of suture remained after cutting. Similarly, another 3-0 absorbable suture was used in the center of the anterior wall, define as point A (Fig. 2B). According to the needlepoint, the anastomotic end was divided into the left and right walls. (2) The first assistant pulled the suture line of the anterior wall (point A) to the left and forward with his right hand and pulled the suture line of the posterior wall (point P) to the right and backward so that the right wall of the anastomosis became the “anterior wall.” The operator used a full-thickness suture of the “front wall” from the pulling point A with a needle distance of 0.5–0.7 cm to avoid excessive tightening (Fig. 2C). The barbed wire was cut when the 3-0 barbed wire was stitched to the suture line at point P to complete the “front wall” suture (Fig. 2D). (3) The suture on the posterior wall (point P) was pulled to the left front, and the suture on the anterior wall (Point A) was pulled to the right rear so that the left wall at the esophagojejunostomy end became the front wall. The full-thickness suture of the right wall was performed from the point P suture (Fig. 2E) with a needle spacing of 0.5–0.7 cm until it coincided with point A. The left wall suture was then completed, as well as the full-thickness suture (Fig. 2F). (4) Next, we continued to pull along the left front of point P and right rear of point A, and the assistant’s right hand could adjust the traction direction appropriately. The 3-0 barbed wire was used to complete the left parietal muscularis enhanced embedding suture (Fig. 2G). After rotating clockwise the same way, the 3-0 barbed wire was used to complete the right parietal muscularis-enhanced embedding suture (Fig. 2H). In order to better show our surgical process, we showed a surgical video in Video clip. The anastomotic time is defined as the time from the beginning of the first suture to the end of the last suture.

### Observation indicators

Basic data of the two groups were collected and compared, including operation time, distance from the tumor to the cutting edge, distance from the jejunal opening to the stump, whether to disconnect the two vascular arches of the jejunum, time of esophagojejunostomy, number of lymph nodes cleaned, amount of intraoperative bleeding, time of first water intake, time of first exhaust, time of hospitalization, postoperative anastomotic complications, and other complications. The severity of anastomotic complications was evaluated by the Clavien-Dindo classification system [11].

### Statistical analysis

The SPSS 22.0 statistical software was for data analysis. Quantitative data are expressed as means ± standard deviations (x̅ ± s). Two independent sample *t* tests were used to compare means between groups. Counting data were analyzed by the *χ*^2^ test or Fisher exact probability method. The confidence level was α = 0.05.

## Results

### Comparison of general data and intraoperative conditions between the two groups

**Fig. 3:**
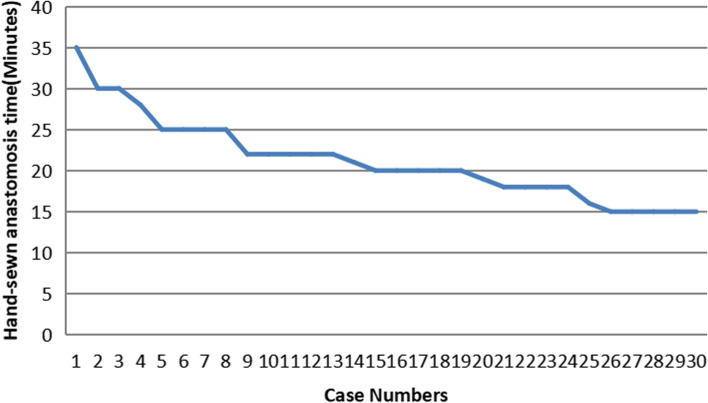
Learning curve

The comparison of general data between the two groups is presented in Tables 1 and 2. The median follow-up time was 18 months for patients in the mechanical group and 22 months for the hand-sewn anastomosis group but did not statistically differ. No significant difference was detected in the overall operation time between the two groups, but the anastomosis time in the manual suture group (21.2 ± 4.9 min) was shorter than that in the mechanical anastomosis group (27.9 ± 6.9 min) (*p* < 0.001). The operating times of manual suture achieved the mean time from the ninth case (Fig. 3). The distance of tumor margin in the manual suture group was statistically longer (2.7 ± 0.4 cm) compared to the mechanical anastomosis group (*p* = 0.002). During the jejunoesophageal anastomosis, the distance between the jejunal opening and the jejunal stump in the manual suture group was significantly shorter (2.2 ± 0.54 cm). Meanwhile, the number of patients needing to disconnect two vascular arches in the mechanical anastomosis group was higher but did not statistically differ (*p* = 0.506). Other indexes, such as the number of lymph node dissections, intraoperative bleeding, the first water intake, the first exhaustion time, and the length of hospital stay, did not significantly differ between the two groups (all *p* > 0.05).

**Table 1:** Comparison of clinical pathology between the two groups (*: using Fisher exact probability method. a Anemia is defined as male HB ≤ 120g/L and female HB ≤ 110 g/L. b Hypoalbuminemia is defined as ≤ 35 g/L)

Clinicopathological features	Hand-sewn group (***n*** = 30)	Mechanical group (***n*** = 30)	Statistical value	***P*** value
**Age(year, x±s)**	**60.6 ± 9.5**	**63.3 ± 8.4**	***t*= 0.326**	**0.570**
**Sex**			***χ***^**2**^**= 1.763**	**0.184**
**Male**	**21**	**16**		
**Female**	**9**	**14**		
**BMI(kg/m**^**2**^**, x ±s)**	**24.2 ± 3.2**	**23.7 ± 2.9**	***t*= 0.205**	**0.653**
**Smoking**			***χ***^**2**^**= 0.617**	**0.432**
**Yes**	**19**	**16**		
**No**	**11**	**14**		
**Alcohol drinking**			***χ***^**2**^**= 0.069**	**0.793**
**Yes**	**12**	**13**		
**No**	**18**	**17**		
**Hypertension**			***χ***^**2**^**= 2.256**	**0.233**
**Yes**	**5**	**10**		
**No**	**25**	**20**		
**Diabetes**			***χ***^**2**^**= 0.418**	**0.748**
**Yes**	**7**	**5**		
**No**	**23**	**25**		
**Combined anemia**^**a**^			***χ***^**2**^= 3.433*	**0.145**
**Yes**	**2**	**7**		
**No**	**28**	**23**		
			***χ***^**2**^**= 2.052**	**0.152**
**Combined with Hypoproteinemia**^**b**^				
**Yes**	**6**	**11**		
**No**	**24**	**19**		
**Siewert classification**			***χ***^**2**^**= 0.084**	**0.347**
**II**	**5**	**8**		
**III**	**25**	**22**		
**Pathological stage**			***χ***^**2**^= 1.700*	**0.427**
**I**	**5**	**4**		
**II**	**13**	**9**		
**III**	**12**	**17**

**Table 2:** Comparison of intraoperative and postoperative recovery between the two groups

Clinical parameters	Hand-sewn group (*n* = 30)	Mechanical group (*n* = 30)	Statistical value	*P* value
Operation time (min, $\overline x\pm s$)	269.8 ± 40.9	272.8 ± 43.5	*t* = 0.141	0.708
Distance between tumor and proximal margin (cm, $\overline x\pm s$ )	2.7 ± 0.44	2.2 ± 0.75	*t* = 3.221	0.002
Distance from jejunal opening to stump (cm, $\overline x\pm s$)	2.2 ± 0.54	5.7±0.6	*t* = − 22.081	< 0.001
Disconnect the two vascular arches of jejunum			*χ*^2^=1.012	0.506
Yes	4	7		
No	26	23		
Anastomosis time(min, $\overline x\pm s$)	21.2 ± 4.9	27.9 ± 6.9	*t* = − 4.349	< 0.001
Number of lymph nodes removed ($\overline x\pm s$)	30.8 ± 3.6	32.9 ± 4.6	*t* = − 1.983	0.052
Blood loss (ml, $\overline x\pm s$)	116.7 ± 68.6	107.7 ± 54.4	*t* = 0.704	0.405
Liquid diet (days, $\overline x\pm s$)	1.2 ± 0.4	1.4 ± 0.5	*t* = − 1.433	0.157
First flatus (days, x±s)	2.4 ± 0.5	2.3 ± 0.5	*t* = 1.064	0.292
Postoperative hospital stay(days, $\overline x\pm s$)	6.6 ± 1.5	6.7 ± 2.6	*t* = 0.785	0.379
Surgery-related costs (CNY, $\overline x\pm s$)	70608.3 ± 8106.7	76485.6 ± 3149.9	*t* = − 3.702	0.001

### Comparison of anastomotic complications and other complications between the two groups

There was no anastomotic bleeding in the observation group after operations. One case in the manual suture group had delayed anastomotic leakage (Clavien IIIa) and was discharged from the hospital 7 days after the operation. The patient was hospitalized for fever due to anastomotic leakage on the ninth day after the operation. The patient was cured and discharged after conservative treatment such as flushing, drainage, and increasing enteral nutritional support. Three cases had fever symptoms (all Clavien I), and the maximum temperature exceeded 38.5 °C, further confirmed as pulmonary infection. They were cured after symptomatic anti-infection treatment. One patient had anastomotic stenosis (Clavien II), which was spontaneously relieved without gastroscopic expansion 3 months after the operation. Three patients (all Clavien II) in the mechanical group presented anastomotic leakage, no fever, abdominal pain, abdominal distension, nausea and vomiting, and abnormal drainage tube volume on the sixth day after the operation. They were cured within 2 weeks after operation by oral full flow slag-free diet and increasing nasal catheter enteral nutrition support. Four patients (all Clavien I) presented fever symptoms. Two cases were confirmed as pulmonary infection, and one with an unknown cause. These patients were symptomatically treated and discharged. The other patient developed high fever symptoms 3 days after discharge (10 days after the operation), with a maximum temperature of 39 °C, accompanied by chills and discomfort. He was admitted to the hospital for a second time and improved the examination. The cause was unclear, and he was discharged after symptomatic treatment (Table 3).

**Table 3:** Comparison of postoperative complications (Fisher exact probability method)

Classification of complications	Hand-sewn group (***n*** = 30)	Mechanical group(***n*** = 30)	Statistical value	***P*** value
**Anastomotic leakage**	**1**	**3**	***χ***^**2**^**= 1.118**	**0.612**
**Anastomotic bleeding**	**0**	**0**	**-**	**-**
**Anastomotic stenosis**	**1**	**0**	***χ***^**2**^**= 1.403**	**1.000**
**Fever**	**3**	**4**	***χ***^**2**^**= 0.162**	**1.000**
**Pulmonary infection**	**3**	**2**	***χ***^**2**^**= 0.220**	**1.000**
**Readmission within 30 days**	**1**	**1**	***χ***^**2**^**= 0.000**	**1.000**
**Clavien-Dindo classification**			***χ***^**2**^**= 1.861**	**0.394**
**Clavien I**	**3**	**4**		
**Clavien II**	**1**	**3**		
**Clavien IIIa**	**1**	**0**		

## Discussion

With the increasing incidence of adenocarcinomas of the esophagogastric junction worldwide, developing TLTG with minimal invasiveness is essential. However, esophagogastric anastomosis in TLTG remains a significant clinical challenge [12]. At present, mechanical anastomosis is widely used in TLTG for gastric cancer. Even the circular stapler technique does not require the exposure of a large portion of the esophagus, and the blood supply of the anastomosis is sufficient, especially on the side of the esophageal stump, which can still be disconnected at a relatively high position [7]. However, it is still rarely used in TLTG, mainly because it is difficult to insert the anvil in the esophageal cavity under endoscopy and is easily affected by the patient’s body size. When using the linear stapler, it is necessary to free at least 5 cm of the healthy esophageal stump before the side-to-side esophagojejunostomy is feasible. In principle, the criteria for successful reconstruction of esophageal jejunal anastomosis include sufficient blood supply and tension-free anastomosis. Nevertheless, too much free esophagus will affect the blood supply of the anastomosis. At the same time, the linear stapler is needed to cut the esophagus longitudinally for 3–5 cm, which will aggravate the blood supply of the esophageal stump. After the anastomosis is completed, the anastomotic stoma will generally retract to the mediastinum, and its tension distribution will be unbalanced. Meanwhile, TLTG has been promoted by the increasing incidence of tumors at the esophagogastric junction, especially for Siewert II patients, due to their lower esophageal availability, high anastomotic position, and difficult mechanical anastomosis, which can easily cause inaccurate anastomosis and increase the risk of anastomotic leakage. In some cases, the anastomosis needs to be resected after the failure of the mechanical anastomosis. Thus, hand-sewn anastomosis becomes an important option for re-anastomosis. Therefore, mechanical anastomosis is not optimal for patients with high lesion locations, considering the high tension, blood supply, and proximal margin safety. In contrast, hand-sewn anastomosis does not need a long esophageal stump and is easy to inspect. Herein, in the manual suture group, the operator tended to resect more esophagus to ensure that the margin was negative while ensuring that no tension could reduce the disconnection of the small intestinal vascular arch and the risk of small intestinal ischemia. During suture, the whole process could be viewed directly to avoid the hidden injury of the machine to the esophagus and jejunum. Furthermore, for the high position or invasive tumors, mechanical anastomosis has higher requirements for the length of the esophageal broken end and jejunum, and the cutting edge is often reserved when leaving the broken esophagus. On the other hand, the hand-sewn suture does not require a high esophageal length, so the average distance between tumor margins is longer. Therefore, we recommend hand-sewn sutures for patients with possible tension of linear anastomosis. In the present study, during jejunoesophageal anastomosis, the distance between the jejunal opening and the jejunal stump in the hand-sewn suture group was significantly shorter, which is particularly important for patients with obesity and short mesentery of the small intestine. For early GEJ cancer, proximal gastrectomy and double flap esophagogastrostomy is a good choice, however, in our retrospective study, the surgeon has chosen total gastrectomy according to his own experience and current consensus, so the advantages of manual suture in proximate gastronomy and double flap esophagogastrostomy are not discussed. However, from a technical point of view, the improvement of this technology may help the operators to improve their experience in manual suture and is more conducive to their later development of totally laparoscopic proximate gastronomy and double flap esophagogastrostomy.

Hand-sewn is considered more time-consuming and challenging to learn than mechanical anastomosis [13]. Our team has performed total laparoscopic gastrectomy for 10 years. Some of them have performed about 800 and 2000 gastric cancer operations respectively. Because we often use barbed wire to close the common opening in gastric colorectal cancer, we have accumulated a lot of experience in laparoscopic surgical anastomosis, so esophagojejunostomy is relatively easy for us. This learning curve may varies greatly depending on the amount of surgery performed in each center. The common opening in esophagojejunostomy and duodenal stump was routinely sutured with barbed wire during total laparoscopic surgery for gastric cancer, so based on our experience, skilled suture can be achieved average time after completing about 9 cases.

Additionally, the requirements for assistants are lower. For example, as long as the suture on both sides was pulled at a fixed angle, the operator could use barbed suture for continuous full-thickness suture from left to right, so the average operation time was shorter than in the mechanical group.

Moreover, the 30 hand-sewn esophagojejunostomy was accomplished except for one grade 2 anastomotic leakage case. This patient was the fifth case of hand-sewn esophagojejunostomy. We believe that the barbed suture damaged the esophageal wall at the beginning of the exercise due to improper strength and direction. Hence, we suggest that the barbed suture should not be excessively tightened, which requires experience from a certain number of cases. Thus, attention should be paid to the barbed wire strength and direction of pulling and the needle distance grasp, especially the needle entry distance of the mucosal layer. Otherwise, it may cause anastomotic stenosis, incomplete anastomosis, poor appearance, and other consequences.

In summary, our current method has the following advantages: (1) it is simple and convenient, with full-thickness suture with barbed thread, which does not affect the blood supply and is conducive to the healing of anastomotic stoma; (2) it can increase the margin distance and reduce the risk of positive margin; (3) less proximal jejunal distance is required to reduce the risk of anastomotic tension; (4) it is still applicable when there is a high risk of machine anastomoses, such as a high tumor, intestinal wall thickening, and edema; (5) the anastomosis time is significantly reduced.

However, our current study also has limitations. The number of patients was small and led to a significant bias. Thus, further multicenter randomized controlled trials are needed to confirm the safety and effectiveness of the new manual suture method.

In conclusion, we provided a new anastomotic method as a choice for surgeons, which may benefit from specific patient populations. Experienced surgeons can try to carry it out, even if its safety and effectiveness still need further verification.

## Supplementary Information

**Additional file 1.**

## Data Availability

All data generated or analyzed during this study are included in this published article.

## Abbreviations

TLTG: Total laparoscopic total gastrectomy
AEG: Adenocarcinoma of the esophagogastric junction
